# Proteomic analysis of primary duck hepatocytes infected with duck hepatitis B virus

**DOI:** 10.1186/1477-5956-8-28

**Published:** 2010-06-07

**Authors:** Yanfeng Zhao, Haijing Ben, Su Qu, Xinwen Zhou, Liang Yan, Bin Xu, Shuangcheng Zhou, Qiang Lou, Rong Ye, Tianlun Zhou, Pengyuan Yang, Di Qu

**Affiliations:** 1 Key Laboratory of Medical Molecular Virology of Ministries of Education and Health, Institute of Medical Microbiology and Institutes of Biomedical Sciences, Shanghai Medical College of Fudan University, Shanghai, China; 2 Department of Chemistry, Institutes of Biomedical Sciences, Fudan University, Shanghai, China

## Abstract

**Background:**

Hepatitis B virus (HBV) is a major cause of liver infection in human. Because of the lack of an appropriate cell culture system for supporting HBV infection efficiently, the cellular and molecular mechanisms of hepadnavirus infection remain incompletely understood. Duck heptatitis B virus (DHBV) can naturally infect primary duck hepatocytes (PDHs) that provide valuable model systems for studying hepadnavirus infection *in vitro*. In this report, we explored global changes in cellular protein expression in DHBV infected PDHs by two-dimension gel electrophoresis (2-DE) combined with MALDI-TOF/TOF tandem mass spectrometry (MS/MS).

**Results:**

The effects of hepadnavirus infection on hepatocytes were investigated in DHBV infected PDHs by the 2-DE analysis. Proteomic profile of PDHs infected with DHBV were analyzed at 24, 72 and 120 h post-infection by comparing with uninfected PDHs, and 75 differentially expressed protein spots were revealed by 2-DE analysis. Among the selected protein spots, 51 spots were identified corresponding to 42 proteins by MS/MS analysis; most of them were matched to orthologous proteins of *Gallus gallus*, *Anas platyrhynchos *or other avian species, including alpha-enolase, lamin A, aconitase 2, cofilin-2 and annexin A2, etc. The down-regulated expression of beta-actin and annexin A2 was confirmed by Western blot analysis, and potential roles of some differentially expressed proteins in the virus-infected cells have been discussed.

**Conclusions:**

Differentially expressed proteins of DHBV infected PDHs revealed by 2-DE, are involved in carbohydrate metabolism, amino acid metabolism, stress responses and cytoskeleton processes etc, providing the insight to understanding of interactions between hepadnavirus and hepatocytes and molecular mechanisms of hepadnavirus pathogenesis.

## Introduction

The HBV, prototype of the Hepadnaviridae family, is a noncytopathic hepatotropic DNA virus replicating via reverse transcription [1]. More than 350 million individuals are HBV carriers worldwide and over one-third of them develop serious liver diseases such as chronic hepatitis, cirrhosis and primary hepatocellular carcinoma [2]. Major obstacles in HBV research have been the inability of the virus to infect cells in vitro and lack of adequate animal models for HBV infection, though primary human hepatocytes and HepaRG cell line have been used to study HBV infection [3]. Human primary hepatocytes and HepaRG cells can support HBV life cycle, but have limitations in accessibility, reproducibility and low level of HBV replication, and a large amount of input virus was needed to infect low proportion of cells [4-6]. DHBV and woodchuck hepatitis B virus (WHBV) are classified into the family of hepadnaviridae. Thus for hepadnavirus infection primary hepatocytes of ducks (DHBV) and woodchucks (WHBV) are still considered as suitable models for investigating the viral replication and pathogenesis [7,8].

The development of proteomic methods has enabled us to investigate the changes of cellular protein expression at a global scale to reveal virus-host interactions [9-12]. The effect of hepadnavirus replication on the host cells, such as the carcinoma derived hepatocyte lines transfected with the HBV genome, HepaRG cell lines or HBV transgenic mice, have been investigated by using 2-DE analysis [13-15]. In the present study, we intend to utilize the DHBV-PDHs system to explore global protein expression changes during hepadnavirus infection by 2-DE. A total of 75 differentially expressed protein spots were revealed by 2-DE between DHBV infected and uninfected PDHs, and 51 protein spots have been identified by MS/MS analysis. Differential expression of beta-actin and annexin A2 was confirmed by Western blot analysis, and potential roles of some differentially expressed proteins in the viral infection have been discussed.

## Results

### DHBV infection of PDHs

**Figure 1:**
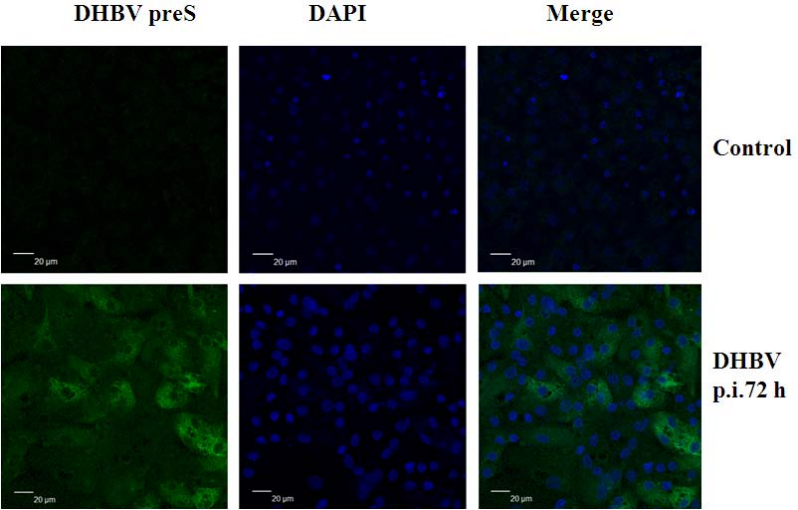
**Detection of DHBV infected PDHs by indirect immunofluorescence**. PDHs were prepared by liver perfusion and seeded on glass coverslips, and infected with DHBV concentrated from the supernatant of LMH-D2 by ultracentrifugation. PDHs were fixed with 4% polyoxymethylene. DHBV infected PDHs was detected by indirect immunofluorescence staining with mouse anti-DHBV preS mAb, and followed by goat anti-mouse IgG-FITC antibody. Then cells were stained with DAPI and visualized by confocal microscopy (Green, FITC; blue, DAPI). The symbol "p.i." represents DHBV post-infection PDHs.

PDHs isolated from the same liver of DHBV-negative Cherry Valley duckling were infected with DHBV at multiplicity of infection of 30 (MOI, based on DHBV DNA genome equivalents) and cultured for 12, 24, 72 and 120 h in L15 medium supplemented with 5% fetal bovine serum (FBS). The efficiency of DHBV infection in PDHs was determined by indirect immunofluorescence using anti-DHBV preS monoclonal antibody. PDHs inoculated with phosphate-buffered saline (PBS, pH 7.2) as a control. At 12 h and 24 h after infection, only a few cells showed fluorescence (data not shown), and at 72 h post-infection about 30% of cells expressed viral large surface antigen, indicating that cells were infected with DHBV, showed in Figure 1. DHBV DNA in PDHs was analyzed by Southern blot hybridization using an alpha-^32^P-dCTP labeled DHBV-specific probe, and DHBV in the supernatant was detected by real time polymerase chain reaction (PCR). Single stranded forms of intracellular viral DNA and DHBV copy number increasing in the supernatant indicated the replication of DHBV 72 h and 120 h post-infection (Additional File 1 and 2).

### 2-DE analysis of differentially expressed proteins of DHBV infected PDHs

**Figure 2:**
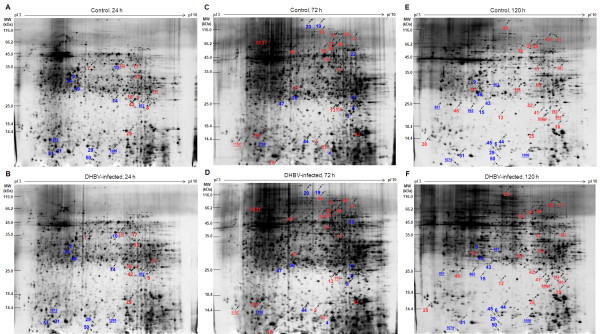
**Comparison of 2-DE gel patterns between DHBV infected and uninfected PDHs**. Proteins were extracted from DHBV infected and uninfected PDHs at 24, 72 and 120 h post-infection. Equal proteins (120 μg) were loaded and separated on the 24-cm pH 3-10 nonlinear IPG strips, followed by 12% SDS-PAGE gels visualized by silver staining. Differentially expressed protein spots on 2-DE gels were identified by MS/MS analysis and shown in gels with unique protein spot numbers. Gels of B, D, F and A, C, E represent 2-DE gels obtained from DHBV infected and uninfected PDHs at 24, 72 and 120 h post-infection, respectively. The numbers of unidentified spots are underlined and listed in Additional File 4, the numbers of up-regulated protein spots in infected samples are labeled in red and the numbers of down-regulated protein spots in blue color (A~F).

Differentially expressed proteins between DHBV infected and uninfected PDHs at 24, 72 and 120 h post-infection were analyzed using the 2-DE. The gels were stained by a modified silver staining method compatible with mass spectrometry (MS) analysis and processed for image analysis. On the 2-DE gels (pH 3-10 NL, 24 cm), about 1150~1350 protein spots were detected. Compared with the parallel uninfected PDHs, 91 differentially expressed protein spots were revealed by 2-DE (*p*-values less than 0.05 with at least a 1.5-fold difference in percentage of the volume), shown in Figure 2 and Additional File 3 (see also Additional File 4), and a total of 75 differentially expressed non-redundant protein spots were analyzed by MS/MS.

### Identification of differentially expressed proteins in DHBV infected PDHs

**Figure 3:**
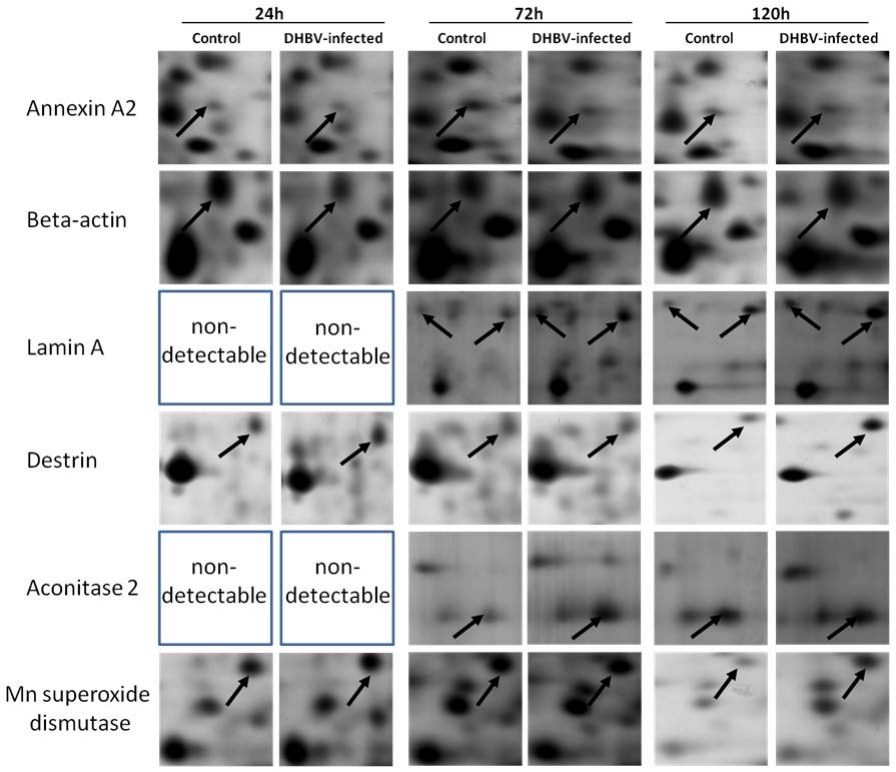
**The enlarged 2-DE profiles of the differentially expressed proteins in the PDHs**. The enlarged images of annexin A2, beta-actin, lamin A, destrin, Aconitase 2 and Mn superoxide dismutase protein spots are shown. Arrows indicate the differentially expressed proteins in 2-DE gels between DHBV infected and uninfected PDHs at 24, 72, and 120 h post-infection.

**Figure 4:**
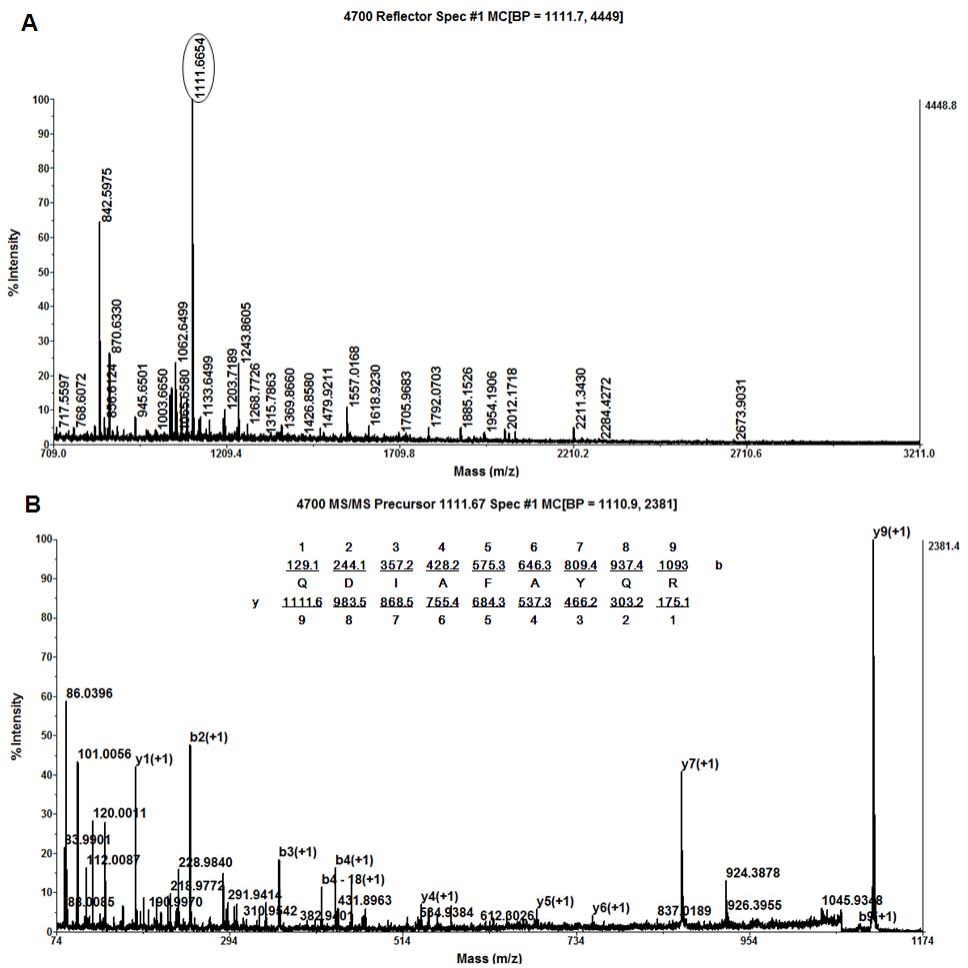
**Representative mass spectra of annexin A2 analyzed by MALDI-TOF/TOF MS**. The differentially expressed protein spot 49 was in-gel digested by trypsin, and peptide mixture was analyzed by MALDI-TOF/TOF Proteomics Analyzer. (A) MS spectrum with tryptic peptides of annexin A2, (B) MS/MS spectrum of the precursor ion with m/z 1111.67 marked in A for peptide QDIAFAYQR of annexin A2; fragments observed in the spectrum were underlined and assigned.

Differentially expressed protein spots between DHBV infected and uninfected PDHs, were excised, digested in-gel with trypsin and determined by MS/MS. Among 75 differentially expressed protein spots, 51 protein spots were identified corresponding to 42 proteins (Table 1 and Additional File 5). With a MASCOT cutoff score of 72 (*p*-value less than 0.05), 51 spots were identified, and 37 spots were matched to orthologous proteins of avian species (26 protein spots to *Gallus gallus*, 4 spots to *Anas platyrhynchos *and 7 spots to other avian species), listed in Table 1. Some of the differentially expressed protein spots such as annexin A2, beta-actin, lamin A, destrin, aconitase 2 and Mn superoxide dismutase were illustrated in enlarged formats (Figure 3), and representative mass spectrum of annexin A2 (spot 49) analyzed by MALDI-TOF/TOF MS was shown in Figure 4. Isoforms of annexin A2, alpha-enolase, lamin A, glyceraldehyde-3-phosphate dehydrogenase (GAPDH), heat shock protein 70 (Hsp70) and elongation factor 2 have been identified. For example, protein spot 49 (MW 31 kDa and p*I *of 5.45) and spot 50 (MW 10 kDa and p*I *of 5.78) down-regulated in DHBV infected PDHs were both identified as annexin A2, and up-regulated protein spot 26 (MW 65 kDa and p*I *of 6.61) and spot 27 (MW 66 kDa and p*I *of 6.4) were matched to lamin A (theoretical MW 73.1 kDa and p*I *of 6.5) showed in Figure 2.

**Table 1:** Differentially expressed proteins in DHBV-infected PDHs identified by MALDI-TOF/TOF

Spot No.^a^	Protein	Species	Accession No.^b^	MOWSE Score^c^	Tmw/Emw^d^	Tp*I*/Ep*I*^e^	Ratio: infected/uninfected		
							
							24h	72h	120h
	**Carbohydrate metabolism**								

1	Triosephosphate isomerase 1	*Gallus gallus*	gi|45382061	385	26.6/29	6.71/6.62	1.5	1	N^f^
2	Triosephosphate isomerase	*Meleagris gallopavo*	gi|34221747	99	22.5/16	6.19/6.00	1.4	3	1
3	Glyceraldehyde-3-phosphate dehydrogenase(GAPDH)	*Columba livia*	gi|6016077	109	35.7/22	8.71/7.41	1	0.5	N
4	Glyceraldehyde-3-phosphate dehydrogenase(GAPDH)	*Columba livia*	gi|6016077	102	35.7/13	8.71/6.23	0.66	0.2	N
5	Glyceraldehyde-3-phosphate dehydrogenase(GAPDH)	*Passer domesticus*	gi|37698402	131	34.8/25	8.71/7.80	1	0.5	N
6	Glyceraldehyde-3-phosphate dehydrogenase(GAPDH)	*Columba livia*	gi|6016077	94	35.7/12	8.71/5.79	1	1	0.2
7	Phosphoglycerate kinase 1	*Gallus gallus*	gi|45384486	231	44.7/42	8.31/5.66	2.5	1.5	1
8	Phosphoglycerate mutase 1	*Gallus gallus*	gi|71895985	135	28.9/25	7.03/7.39	1.5	1.3	1
9	Alpha-enolase	*Anas platyrhynchos*	gi|213085	124	40.8/32	6.28/5.45	0.66	0.8	0.5
10	Alpha-enolase	*Anas platyrhynchos*	gi|119338	340	47.2/43	6.37/6.12	0.25	N	N
11	Aconitase 2, mitochondrial	*Gallus gallus*	gi|45383738	227	85.7/103	8.05/7.78	N	2	1.8
12	ATP5A1	*Taeniopygia guttata*	gi|91805309	380	21.5/23	6.4/5.85	1.2	0.9	2.3
13	Atp5a1 protein	*Xenopus tropicalis*	gi|71896075	146	59.8/24	9.13/6.47	1.3	0.56	1
14	Chain E, Chicken Cytochrome Bc1 Complex Inhibited By An Iodinated Analogue Of The Polyketide Crocacin-D	*Xenopus tropicalis*	gi|196049779	146	21.5/27	6.07/6.00	0.17	N	1
15	similar to ubiquinol--cytochrome c reductase	*Gallus gallus*	gi|50754375	72	52.7/24	6.58/5.45	1	1.2	0.5

	**Amino acid metabolism**								

16	unnamed protein product	*Mus musculus*	gi|74183518	118	35.8/28	6.84/6.80	1.5	1	1
17	similar to betaine homocysteine methyl transferase	*Gallus gallus*	gi|50755288	96	45/44	7.56/6.80	1.5	2	1.5
18	Pterin-4 alpha-carbinolamine dehydratase	*Gallus gallus*	gi|45382483	170	12.0/11	6.04/5.42	1	1.8	N
19	similar to Urocanase domain containing 1	*Gallus gallus*	gi|50754419	127	75.1/149	7.21/6.12	1	0.5	1.2
20	similar to Urocanase domain containing 1	*Gallus gallus*	gi|50754419	99	75.1/149	7.21/5.91	1	0.66	1.7
21	similar to 3-mercaptopyruvate sulfurtransferase	*Gallus gallus*	gi|50794693	144	33.2/32	5.66/5.80	0.75	0.25	N
22	Aldehyde dehydrogenase 4A1	*Xenopus laevis*	gi|148228402	99	61.7/58	8.22/7.18	N	3	1.2

	**Cytoskeletal/structural protein**								

23	Transgelin	*Gallus gallus*	gi|45382783	92	22.3/19	8.85/7.78	1	3	1.4
24	Vinculin	*Gallus gallus*	gi|45382123	82	116.9/116	6/6.12	N	3	1
25	Destrin	*Gallus gallus*	gi|45382979	97	18.5/18	7.52/6.60	1.5	1.33	2
26	Lamin A	*Gallus gallus*	gi|45384214	168	73.1/65	6.5/6.61	N	2	2
27	Lamin A	*Gallus gallus*	gi|45384214	74	73.1/66	6.5/6.40	N	1.5	2
28	similar to Myosin regulatory light chain 2,nonsarcomeric (Myosin RLC) isoform 1	*Canis lupus familiaris*	gi|73961895	241	20.5/15	4.62/3.80	1	1	A/N^g^
29	similar to HSPC162(dynein, light chain)	*Gallus gallus*	gi|50758587	86	10.9/11	6.58/5.79	0.5	1	0.33
30	Collapsin response mediator protein-2B	*Gallus gallus*	gi|33340025	173	62.2/69	6.05/6.12	N	A/N	2
31	Cofilin-2	*Gallus gallus*	gi|17433708	74	18.6/14	7.66/5.20	0.33	1.2	0.67
32	Gelsolin	*Gallus gallus*	gi|45384386	95	85.8/14	5.93/6.19	N	0.25	1
33	similar to LIM protein	*Danio rerio*	gi|68371150	84	35.1/37	6.2/6.61	N/A^g^	1.3	1
34	beta-actin	*Labeo calbasu*	gi|18034011	343	41.7/41	5.16/5.40	0.63	0.8	0.94

	**Stress response**								

35	similar to heat shock 70kDa protein 8 isoform 2 isoform 2	*Canis lupus familiaris*	gi|74012289	74	53.5/20	5.59/7.80	N	N	A/N
36	Chain A, T13g Mutant Of The ATPase Fragment Of Bovine Hsc70	*Bos taurus*	gi|6729825	237	41.9/29	6.63/5.72	0.7	0.33	1
37	Heat shock 70kDa protein 5 precursor	*Gallus gallus*	gi|45382769	526	72/96	5.12/5.00	N	A/N	1
38	Heat shock 70kDa protein 5 precursor	*Gallus gallus*	gi|45382769	471	72/96	5.12/5.00	N	A/N	1
39	Chaperonin containing TCP1, subunit 6A (zeta 1)	*Gallus gallus*	gi|57525300	211	57.6/65	6.36/6.60	1	2	1.3
40	58kDa glucose regulated protein precursor	*Gallus gallus*	gi|45383890	251	56.1/62	5.76/5.69	N	2	1
41	Mn superoxide dismutase	*Cairina moschata*	gi|184133036	235	12.9/24	6.39/7.00	1	1	1.71
42	similar to antioxidant protein isoform 2 (peroxiredoxin-3)	*Sus scrofa*	gi|194042134	256	26.6/25	6.79/6.80	1.5	1.43	1.5
43	LOC496089 protein	*Xenopus laevis*	gi|56269242	97	29.9/28	6.07/5.70	1	1	0.5

	**Other functions**								

44	Elongation factor 2	*Oxyuranus scutellatus*	gi|63146080	83	45.1/16	5.83/5.85	1	0.66	0.67
45	Elongation factor 2	*Mus musculus*	gi|192989	140	29.9/15	6.2/5.78	1	1	0.5
46	Cathepsin B	*Gallus gallus*	gi|46195455	73	37.6/25	5.74/5.08	1	1	1.5
47	ubiquitin carboxyl-terminal esterase L1 (ubiquitin thiolesterase)	*Taeniopygia guttata*	gi|115391986	132	25.2/28	5.83/5.45	N	0.29	0.85
48	Ovotransferrin	*Anas platyrhynchos*	gi|3024757	144	75.6/91	6.19/6.60	N	1.5	1.3
49	Annexin A2	*Gallus gallus*	gi|45382533	148	38.6/31	6.92/5.45	0.33	1	0.5
50	Annexin A2	*Gallus gallus*	gi|45382533	204	38.6/10	6.92/5.78	0.5	N	0.2
51	Annexin A5	*Anoplopoma fimbria*	gi|229366222	75	34.9/12	5.28/5.00	0.5	1	0.86

a) Spots numbers correspond to the numbers in Figure 2.

b) Accession no. is the MASCOT result of MALDI-TOF/TOF searched from the NCBInr database.

c) Protein score was from MALDI-TOF/TOF identification. The proteins that had a statistically significant score great than 72 (*p *< 0.05) were considered identified.

d) Theoretical/experimental molecular mass.

e) Theoretical/experimental molecular p*I*.

f) Not applicable, because the spots on the gels were too weak or non-detectable.

g) A represents the spot on one of the gels was detectable, N represents the spot on one of the gels was too weak to detect.

Biological functions of the differentially expressed proteins in the DHBV-infected PDHs, were analyzed according to the Gene Ontology criteria and classified into carbohydrate metabolism (29%), amino acid metabolism (14%), cytoskeletal/structural protein (24%), stress response (18%) and other functions (16%), as shown in Table 1. The roles of selected differentially expressed proteins reported in viral infections were showed in Table 2.

**Table 2:** The roles of differentially expressed proteins reported in viral infections

Protein Name	Functions indicated in viral infections	References
Aconitase 2, mitochondrial	Binding to the 3'-untranslated region of the mouse hepatitis virus genome and increasing virus production as well as viral protein synthesis at early hours of infection	Nanda et al., 2001. J Virol. 75, 3352-62.
Phosphoglycerate kinase 1	Regulating Sendai virus transcription through their interactions with tubulin	Ogino et al., 2001. Biochem Biophys Res Commun. 285, 447-55.
Triosephosphate isomerase	Increased between cancerous and noncancerous tissues from hepatocellular carcinoma patients	Kuramitsu et al., 2005. Expert Rev Proteomics. 2, 589-601.
Phosphoglycerate mutase 1	Specifically binding to the core region of HCV RNA genome in vitro	Su et al., 2007. Intervirology. 50, 303-9.
Alpha-enolase	A candidate biomarker for HCV-related hepatocellular carcinoma; required for efficient transcription of Sendai virus genome	Takashima et al., 2005. Proteomics. 5, 1686-92. Ogino et al., 2001. Biochem Biophys Res Commun. 285, 447-55.
GAPDH	May interfere in the life-cycle of HBV to phosphorylate HBV core protein and playing roles in HBV infection in intracellular processes	Duclos-Vallee et al., 1998. J Gen Virol. 79 (Pt 7), 1665-70. Duclos-Vallee et al., 2002. Hepatol Res. 22, 65-73.
Lamin A	Impeding HSV-1 infectivity; Phosphorylated in HSV-infected cells, regulating virus capsid nuclear egress; Epstein-Barr virus reactivation-mediated redistribution of nuclear lamin to modulate the cellular environment for virion production; cytomegalovirus induced distortion of the nuclear lamina	Mou et al., 2008. J Virol. 82, 8094-104. Mou et al., 2007. J Virol. 81, 6459-70. Lee et al., 2008. J Virol. 82, 11913-26. Milbradt et al., 2007. J Gen Virol. 88, 2642-50.
Beta-actin	Actin rearrangements contribute to Simian virus 40 endocytosis; required by viral particle up-taking and infection establishment, including HIV, adenovirus, Simian virus 40, and vaccinia virus, etc	Pelkmans et al., 2002. Science. 296, 535-9. Pollard et al., 2003. Cell. 112, 453-65. Bukrinskaya et al., 1998. J Exp Med. 188, 2113-25. Li et al., 1998. J Virol. 72, 8806-12. Ploubidou et al., 2001. Curr Opin Cell Biol. 13, 97-105.
Dynein, light chain	Regulating Gag and viral RNA egress on endosomal membranes in the cytoplasm to directly impact on viral production; microtubule-dependent dynein activity increased through activation of the p38 MAPK by HBx, perhaps facilitating the process of maturation; directly used by poliovirus for retrograde axonal transport	Lehmann et al., 2009. J Biol Chem. 284, 14572-85. Kim et al., 2007. J Virol. 81, 1714-26. Gonzalez et al., 2007. Intervirology. 50, 214-8.
Cofilin-2	Possible involving in the process of HIV initial binding and fusion steps, and inhibiting some subsequent early post-entry events in HIV infection of T cells	Liu et al., 2009. Sci Signal. 2, pe23.
Heat shock protein 70B	HBV P protein activation in vitro is fundamentally dependent on Hsc70/Hsp40; involving in HBV morphogenesis as a chaperone	Beck et al., 2003. J Biol Chem. 278, 36128-38. Prange et al., 1999. Biol Chem. 380, 305-14.
Annexin A2	Assisting in the assembly of HIV, and supporting HIV-1 infection as a cellular cofactor; enhancing cytomegalovirus binding and membrane fusion and supporting the replication of influenza viruses by mediating activation of plasminogen	Ma et al., 2004. J Exp Med. 200, 1337-46. Raynor et al., 1999. Biochemistry. 38, 5089-95. LeBouder et al., 2008. J Virol. 82, 6820-8.
Elongation factor 2	Interacting with hepatitis B virus core protein in leukocytes; possessing a highly conserved anti-apoptotic activity induced by HIV-1 viral protein R	Lin et al., 2006. World J Gastroenterol. 12, 1043-8. Zelivianski et al., 2006. Apoptosis. 11, 377-88.
Ovotransferrin	Playing a crucial role in protecting the whole chicken embryo fibroblasts from Marek's disease virus infection spreading	Giansanti et al., 2007. Biochem Cell Biol. 85, 150-5.

### Validation of differentially expressed proteins

**Figure 5:**
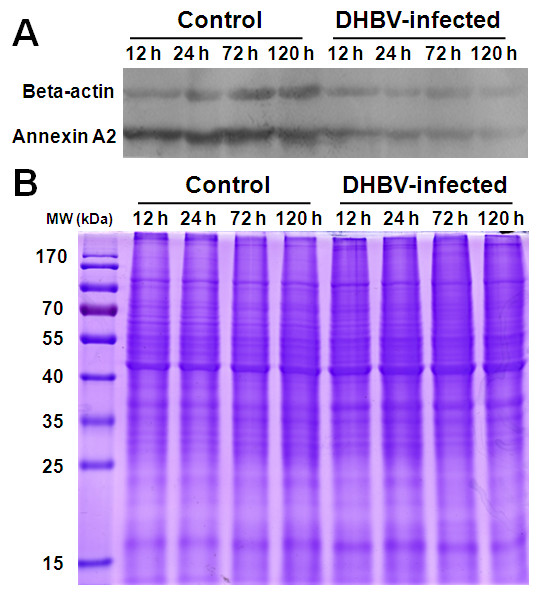
**Confirmation of the differentially expressed proteins of annexin A2 and beta-actin by Western blot**. The equal cell lysates of control and DHBV infected PDHs at 12, 24, 72 and 120 h post-infection were separated by SDS-PAGE, and detected by Western blotting with mouse anti-beta-actin and anti-duck annexin A2 as primary antibodies and then followed by horseradish peroxidase-conjugated goat anti-mouse IgG as secondary antibody (A). The same amount protein of each sample were applied to a parallel SDS-PAGE gel and stained with Coomassie brilliant blue (B).

Expression levels of annexin A2, beta-actin, Hsp70, destrin, and lamin A were validated by Western blot analysis to confirm the dynamic alterations of protein expression during DHBV infection. Equal amounts (30 μg) of cell lysates of DHBV-infected and uninfected PDHs at 12, 24, 72 and 120 h post-infection were separated by SDS-PAGE. Duck beta-actin and annexin A2 expression were detected down-regulated in the DHBV-infected PDHs at 12-120 h post-infection with mouse anti-beta-actin and anti-duck-annexin A2 as primary antibodies (Figure 5A), that were consistent with the protein expression pattern revealed by the 2-DE analysis. Duck Hsp70, destrin, and lamin A were not detected by Western blot analysis with the rabbit anti-human or anti-mouse Hsp70 polyclonal antibodies, rabbit anti-human destrin polyclonal antibody and rabbit anti-human lamin A polyclonal antibody. The same amount protein of each sample were applied to a parallel SDS-PAGE gel and stained with Coomassie brilliant blue (Figure 5B).

## Discussion

HBV infection remains a public health problem worldwide. Because the lack of appropriate cell lines that can support HBV infection efficiently, the cellular and molecular mechanisms of hepadnavirus infection remain incompletely understood. The hepadnavirus animal infection models such as ducks (DHBV) and woodchucks (WHBV) have been used to investigate the viral replication, pathogenesis or hepadnavirus-associated hepatocellular carcinoma. DHBV-PDHs model is a valuable model of hepadnavirus infection with high reproducibility and efficiency [16]. In the present study, global changes in cellular protein expression in DHBV-infected PDHs were explored by 2-DE combined with MS/MS. Among the 75 differentially expressed protein spots, 51 spots have been identified by MS/MS corresponding to 42 proteins, in which 30 spots were matched to orthologous proteins of *Gallus gallus *or *Anas platyrhynchos*, 7 spots to other avian species, and 14 spots to non-avian species, while mass spectra of the other 24 protein spots did not match to any proteins in the current databases, possibly due to the incomplete genome sequence of *Anas platyrhynchos *or low abundance of those protein spots.

In previously studies, Tong performed a proteomic analysis comparing HepG2 with HepG2.2.15 in which HBV genome integrated into cellular chromosome [13], and Narayan revealed 19 differentially regulated features in HepaRG cells by 2-DE [14]. HepG2.2.15 is a HBV replication cell model but not an infection model, while the human hepatoma HepaRG cells are susceptible to HBV, but 10~20% of cells can be infected regardless of the amount of virus used (MOI > 200) [4,6]. In previous studies, it has been showed that at MOI of 30, about 50%~60% PDHs can be reproducibly infected with DHBV [17]. Some of differentially expressed proteins identified in the present study, such as alpha-enolase, lamin A, GAPDH and cofilin-2 have not yet been reported in hepadnavirus proteomic analysis.

Viruses depend on host cell metabolism for their replication. Elucidation of the pathways/processes involving in the viral life cycle will help to understand the mechanisms of viral infection. In the 2-DE analysis, the identified differentially expressed proteins were classified into carbohydrate metabolism, amino acid metabolism, cytoskeletal/structural protein, stress response and other functions according to the Gene Ontology criteria. Some of differentially expressed proteins identified in the present study have been reported playing roles in viral infections, as shown in Table 2.

In DHBV infected PDHs, the expression of some carbohydrate metabolic enzymes, such as phosphoglycerate kinase 1, triosephosphate isomerase, phosphoglycerate mutase 1 etc, was up-regulated. The differentially expressed proteins involving in carbohydrate metabolism, suggests perturbed energy metabolism in DHBV infections. Hepatitis C Virus (HCV) infection reprograms the cellular metabolisms to favor glucose fermentation and glycolytic intermediates toward the metabolite synthesis that supports the viral life cycle [18]. In lymphocytic choriomeningitis virus infection, there was a significant increasing in transcripts promoting gluconeogenesis for viral mediate synthesis, and a decreasing in transcripts promoting glycogenolysis in the early stage of infection [19].

However, GAPDH and alpha-enolase, key enzymes involving in glycolysis and gluconeogenesis, are decreased in DHBV infected PDHs. GAPDH and alpha-enolase have been found associating with the cell membrane and in secreted viral particles of influenza virus, lentiviral vector etc [20,21]. GAPDH may phosphorylate the HBV core protein, and binds to the preS1 region of the HBV envelope antigen and posttranscriptional regulatory element in regulating expression of surface antigen, suggesting that GAPDH plays an important role in the life-cycle of HBV infection [22-24]. The host cellular carbohydrate metabolism affected by DHBV infection may benefit viral replication.

Alterations of cytoskeleton networks were found in many viral infections [25-27]. Hepadnavirus needs to manipulate and utilize the host cytoskeleton to promote viral infection like many viruses, although the mechanism is still unclear [28]. In DHBV infected PDHs, the microfilament-associated proteins, beta-actin and cofilin-2 were down-regulated, and three microfilament-associated proteins such as transgelin, destrin, and collapsin response mediator protein-2B were up-regulated. Actin plays an active role in maturation of the viruses [29,30]. Many viruses require actin for viral entry and establishment of infection, including human immunodeficiency virus (HIV), adenovirus, Simian virus 40, and vaccinia virus [31-34]. However, the actin cortex beneath the plasma membrane can also be an obstacle for virus entry or budding [35]. It has been reported that DHBV entry depends on both intact microtubules and their dynamic turnover but not actin cytoskeleton [28]. Therefore the role of actin in DHBV replication is required to further investigation.

Lamin A is key structural components of the nuclear lamina and lamins, involving in DNA replication and gene expression, as well as presenting a natural barrier against most DNA viruses such as human cytomegalovirus (HCMV), Kaposi's sarcoma-associated herpesvirus, herpes simplex virus (HSV) 1 and Epstein-Barr virus [36]. Lamin A/C is phosphorylated in HSV-infected cells supporting a role in regulating virus capsid nuclear egress [37,38]. Infection of Epstein-Barr virus induced disassembly of the nuclear lamina and redistribution of nuclear lamin for the nuclear egress [39]. The expression of lamin A with different isoforms, were up-regulated in DHBV infected PDHs, suggesting that lamin A may play a role in DHBV replication.

In DHBV infected PDHs, up-regulated expressions of amino acid metabolism enzymes, catalyzing interconversion of glutamate, histidine, and proline (Glutamate dehydrogenase 1, Urocanate hydratase, Delta-1-pyrroline-5-carboxylate dehydrogenase, the orthologs in human referred to protein 16, 19 and 20, 22 in Table 1), indicate that glutamine metabolism is enhanced. Switching the anaplerotic substrate from glucose to glutamine to accommodate the biosynthetic and energetic needs of the viral infection and to allow glucose to be used biosynthetically was reported in HCMV infection [40]. HCV-infected cells exhibit increased levels of the enzymes catalyzing glutamine flux to replenish metabolic intermediates through the latter half of the citric acid cycle providing substrates for ATP production [18]. Thus similar mechanism of glutamine metabolism may be at work in DHBV infection.

Stress response associated proteins including endoplasmic reticulum stress associated proteins such as Hsp70, and chaperonin containing t-complex polypeptide 1 (TCP1) and oxidative stress associated proteins such as antioxidant enzymes Mn superoxide dismutase and peroxiredoxin-3 (similar to antioxidant protein isoform 2) were found to be up-regulated post DHBV infection. Hsp70 assists folding of many newly synthesized polypeptides, and refolding of the proteins misfolded [41,42]. Hsp70 can enhance flock house virus replication [43,44]. Hsp70 and Hsp90 participate in dengue virus entry as a receptor complex [45]. Moreover, HBV P protein activation in vitro is fundamentally dependent on heat shock protein 70 family Hsc70/Hsp40 [46]. In HBV-replicating HepAD38 cell, expressions of heat shock proteins (Hsp70 and Hsp90) and Mn superoxide dismutase increase, after HBV replication induced by tetracycline [47]. In humanized transgenic mice, inhibition of HBV replication results in suppression of Mn superoxide dismutase expression in hepatocytes [47,48]. It suggests that oxidative stress can be induced by hepadnavirus replication as Epstein-Barr virus [49].

Annexin A2, belongs to a family of calcium-dependent, phospholipid binding proteins, is involved in many biological processes, such as the Ca^2+ ^dependent exocytosis, calcium transport and cell proliferation. It participates in viral infection, including assisting in the assembly of HIV in monocyte-derived macrophages [50], as a cellular cofactor supporting HIV-1 infection [51], enhancing cytomegalovirus binding and membrane fusion [52] and supporting the replication of influenza viruses by mediating activation of plasminogen [53]. It has been reported that HBV polymerase activity was inhibited by interacted with S100A10, a protein binding to annexin A2 [54]. In HepG2.2.15 compared with HepG2, annexin A2 was revealed down-regulated [55], which was consistent with our observation in DHBV-PDHs model and confirmed by Western blot analysis. It indicated that annexin A2 may involve in hepadnavirus infection and warrants further investigation.

Beta-actin and GAPDH are usually referred as the internal standards for detections of RNA transcription and protein expression of genes. However, those proteins were found to be down-regulated post DHBV infection by 2-DE analysis. Recently, accumulated evidence showed that in HBV-related hepatocellular carcinoma or viral infections, beta-actin and GAPDH are unsuitable controls in quantitative mRNA expression or Western blot analysis due to variations in expression [56-60], though there are controversial observations [61]. These findings therefore highlight the importance of re-evaluating the housekeeping genes whose expressions may be affected by hepadnavirus infection.

## Conclusions

In summary, the present study explored global changes in cellular protein expression of hepadnavirus infection by 2-DE analysis, using a natural DHBV-PDHs infection system. Forty-two differentially expressed proteins in DHBV infected PDHs have been identified by MS/MS. Most of them involve in carbohydrate metabolism, amino acid metabolism, stress responses and cytoskeleton processes including alpha-enolase, beta-actin, lamin A and annexin A2. It suggests that those proteins may play important roles in hepadnavirus infection. Differential expressions of annexin A2 and beta-actin were confirmed by Western blot analysis. Further investigation of the roles of the differentially expressed cellular proteins will help to understand cellular and molecular mechanisms of hepadnavirus infection.

## Materials and methods

### PDHs culture

Cherry Valley ducks (*Anas platyrhynchos*) were purchased from Breeding Center of Shanghai Institute of Veterinary Medical Sciences, China. Animal protocols were approved by the Ethics Committee of Fudan University. Three-day-old ducklings with no congenital DHBV infection detected by PCR (sence: 5'-CTCACTTTGTGGATCTCATTG-3', antisense: 5'-ATCGGATAGTCGGGTTGG-3'), were used for PDHs cultures. Duck hepatocytes were isolated with in situ liver perfusion method with modifications. After the duck was anesthetized with approximately 0.3 ml of 0.75% pentobarbital sodium, the liver was perfused via portal vein with pre-warmed liver perfusion medium (Gibco Laboratories), then the inferior vena cava was cut to effuse the buffer liquid when the liver was engorged. At first, perfusion was maintained at 15 to 20 ml *per *minute with 100 ml of liver perfusion medium until the liver became blanch, then the liver became soft followed by 50 ml digestion buffer with 1 μg/ml of collagenase type IV (Sigma) in L15 medium (Gibco). After the perfusion, the gallbladder was removed and hepatocytes were dispersed in L-15 medium. Hepatocytes were filtered through sterilized gauze, centrifuged at 40 *g *for 4 min, and washed three times with Hepatocyte Wash Medium (Gibco). Then 4 × 10^6 ^hepatocytes were seeded onto 100-mm-diameter dish and incubated in L-15 medium containing 5% FBS (Gibco Laboratories), 15 mM HEPES, 100 U penicillin *per *liter, 100 mg of streptomycin *per *liter, 1 mg of insulin (Sigma) *per *liter and 10^-5 ^M hydrocortisone-hemisuccinate (Sigma) at 37°C. The medium was changed every day.

### DHBV infection of PDHs

Infectious DHBV were produced by LMH-D2 cell line which carries a stably integrated DHBV dimer and constitutively secretes DHBV virions (generous gift of William Mason, Fox Chase Cancer Center, USA) [62]. DHBV particles were obtained from LMH-D2 cells by ultracentrifugation, and the virus pellet was suspended in PBS with 10% glycerol. DHBV was quantified using real time PCR as a titer of 2 × 10^9 ^copies *per *milliliter. PDHs cultured 16 h after plating were infected with purified DHBV viral particles at MOI of 30, and incubated at 37°C overnight. Then, they were washed with PBS three times and cultured for 12, 24, 72 and 120 h in L15 medium supplemented with 5% FBS.

### Detection of DHBV infected PDHs

The efficiency of DHBV infection in PDHs was determined by indirect immunofluorescence and Southern blot hybridization. Monolayers of PDHs grown on glass coverslips were fixed directly by adding 4% polyoxymethylene at room temperature for 20 min, washed twice with PBS and preincubated with 3% bovine serum albumin for 30 min. After incubation with a 1:100 dilution of monoclonal mouse anti-DHBV preS (generous gift of John C. Pugh and William Mason, Fox Chase Cancer Center, USA) at 37°C for 60 min, the cells were washed three times with PBS, subsequently incubated with a 1:200 dilution of FITC-conjugated sheep anti-mouse IgG (GGHL-90F, Immunology Consultants Laboratory) at 37°C for 30 min. Cell nucleus were stained with 1 μg/ml 4',6'-diamidino-2-phenylindole (DAPI, Sigma) and mounted in 50% glycerol in PBS. Efficiency of DHBV infection was observed by the confocal laser scanning microscope (Leica). To detect DHBV replication, intracellular DNA was extracted from DHBV infected or uninfected PDHs. Forty micrograms of DNA from each sample was separated on a 1.5% agarose gel, and analyzed by Southern blot hybridization with an alpha-^32^P-dCTP labeled DHBV-specific probe for detection as described previously [63].

### PDHs' protein sample preparation for 2-DE

The DHBV-infected and control (uninfected) PDHs at 24, 72, and 120 h post-infection were washed three times with ice-cold PBS before harvesting and stored at -80°C. PDHs for DHBV infection or the uninfected controls were from the same duck in order to avoid individual differences. Approximately 2 × 10^7 ^cells were lysed in 1 ml lysis buffer (7 M urea, 2 M Thiourea, 2% (w/v) CHAPS, 50 mM dithiothreitol (DTT), 2% (v/v) pH 3-10 nonlinear immobilized pH gradient (IPG) buffer (Amersham Biosciences) containing 1% protease inhibitor cocktail (Roche) and 1 mM PMSF (Sigma)), then sonicated on ice for 12 cycles, each consisting of 5 s pulse and 10 s pause. After centrifugation at 20,000 *g *at 4°C for 1 h, the supernatants of lysates were divided into aliquots and the protein concentrations were determined by the Bradford assay. Then, aliquots were stored at -80°C for further analysis.

### Two-dimensional gel electrophoresis and image analysis

The 2-DE gels were performed using 24-cm IPG strips (pH 3-10, nonlinear, GE Healthcare) in Ettan IPGphor Isoelectric Focusing System (Amersham Biosciences) plus Ettan-Dalt six system (Amersham Biosciences) according to the manufacturer's instructions. To compensate the variability of gel electrophoresis, at least three replicate gels were performed for each group. In the first dimensional isoelectric focusing (IEF), 120 μg proteins of each sample were diluted to 450 μl with rehydration buffer containing 8 M urea, 2% (w/v) CHAPS, 50 mM DTT, 0.5% (v/v) ampholyte (pH 3-10, nonlinear, Amersham Biosciences), and IPG strips were allowed to rehydrate in the above solution under mineral oil. IEF was performed as follow: 30 V for 6 h (active rehydration); 60 V for 6 h (active rehydration); 500 V for 2 h, rapid; 1,000 V for 2 h, rapid; 4,000 V for 2 h, linear; linear ramping to 8,000 V for 2 h, and finally 8,000 V for about 7 h with a total of 64 KVh at 20°C. Then the IPG strips were incubated in equilibration buffer (75 mM Tris-HCl (pH 8.8), 6 M urea, 29.3% (v/v) glycerol, 2% (w/v) SDS and 0.002% (w/v) bromophenol blue) containing 2% (w/v) DTT for 15 min with gentle agitation, followed by incubation in the same equilibration buffer supplemented with 2.5% (w/v) iodoacetamide for 15 min at room temperature. The second dimension SDS-PAGE was performed on 1 mm thick 12.5% polyacrilamide vertical gels in Ettan-Dalt Six system using 5 W/gel for 30 min, and followed by 12 W/gel at 10°C until the bromophenol blue dye front reached the end of the gels. The gels were stained by a modified silver staining method compatible with MS analysis [64] and scanned at 300 dpi (dots/inch) using ImageScanner (UMAX, Amersham Biosciences).

Images were captured and analyzed by ImageMaster 2D platinum 6.0 software (Amersham Biosciences). The percentage of the volume of the spots representing a certain protein was determined in comparison with the total proteins present in the 2-DE gel. To select differentially expressed protein spots, quantitative analysis was performed using the Student's *t*-test to compare the percentage volumes of spots between DHBV infected and uninfected groups at three time points. The differentially expressed protein spots with *p*-values less than 0.05 were considered as significant differences, and at least 1.5 fold difference in percentage of the volume for each spot was set as a threshold. These protein spots were selected and subjected to in-gel tryptic digestion and identification by MS.

### In-gel tryptic digestion

The differentially expressed protein spots were manually excised from the sliver-stained gels (each gel of 120 μg protein) and placed into a 96-well microplate. The gel pieces were destained with a solution of 15 mM potassium ferricyanide and 50 mM sodium thiosulfate (1:1) at room temperature for 10 min, then washed twice with deionized water, each for 30 min, and dehydrated in 80 μl of acetonitrile (ACN) twice. Then the samples were swollen in a digestion buffer containing 25 mM NH_4_HCO_3 _and 12.5 ng/μl trypsin (Promega) at 4°C after 30 min incubation, and incubated at 37°C for more than 12 h. The peptide mixtures from the gel were extracted twice using 0.1% trifluoroacetic/50% ACN at room temperature, re-suspended with 0.7 μl matrix solution (α-cyano-4-hydroxy-cinnamic acid (Sigma) in 0.1% trifluoroacetic, 50% ACN), allowed to dry in air under the protection of N2.

### Mass spectrometric analysis and database searching

The peptide mixtures from samples were analyzed by 4700 MALDI-TOF/TOF Proteomics Analyzer (Applied Biosystems). The UV laser was operated at a 200 Hz repetition rate with wavelength of 355 nm. The accelerated voltage was operated at 20 kV. Myoglobin digested by trypsin was used to calibrate the mass instrument with internal calibration mode. All acquired spectra of samples were processed using 4700 series Explore software (Applied Biosystems) in a default mode. The parent mass peaks with mass range 700-3200 Da and minimum S/N 20 were picked out for tandem TOF/TOF analysis. Combined MS and MS/MS spectra were submitted to MASCOT (V2.1, Matrix Science) by GPS Explorer software (V3.6, Applied Biosystems) and searched with the following parameters: NCBInr database (release date: 2009.11), taxonomy of bony vertebrates or viruses, trypsin digest with one missing cleavage, none fixed modifications, MS tolerance of 100 ppm, MS/MS tolerance of 0.6 Da and possible oxidation of methionine. Known contaminant ions (human keratin and tryptic autodigest peptides, etc.) were excluded. MASCOT protein scores (based on MS and MS/MS spectra) with greater than 72 were considered statistically significant (*p *< 0.05). The individual MS/MS spectrum with statistically significant (confidence interval >95%) and best ion score (based on MS/MS spectra) was accepted. To eliminate the redundancy of proteins that appeared in the database under different names and accession numbers, the protein belonging to the species *Anas platyrhynchos *or with the highest protein score (top rank) was singled out.

### Preparation of polyclonal mouse anti-annexin A2

Duck cDNA of annexin A2 was amplified by reverse transcription-PCR with the primers designed according to the sequence of the chicken annexin A2 (GenBank Accession No. gi|45382533, sense: 5'-CCGCTCGAGGTCCTCTCCACCACACAGGT-3' and antisense: 5'-CCGCTCGAGGTCCTCTCCACCACACAGGT-3'). The full-length of duck annexin A2 amplified from PDHs, was cloned into prokaryotic expression plasmid pET-28a (Novagen). Recombinant annexin A2 with C-terminal fusion His-tag was induced by IPTG, and purified by Ni-NTA affinity chromatography (QIAGEN). BALB/c mice were immunized by the purified recombinant duck annexin A2 with Freund's complete adjuvant (Sigma). The serum was collected 2 weeks following the final injection and the levels of anti-duck-annexin A2 antibody titers from immunized mice were determined by Western blot.

### Western blot analysis of differential proteins

Differential expression of duck beta-actin, annexin A2, Hsp70, destrin and lamin A were confirmed by Western blot analysis. The primary antibodies for detection were as follow: a monoclonal antibody against beta-actin (Sigma), rabbit anti-human lamin A polyclonal antibody (Santa Cruz Biotechnology and Proteintech), rabbit anti-human Hsp70 polyclonal antibody (Santa Cruz Biotechnology), rabbit Hsp70 polyclonal antibody (BIOS), rabbit anti-human destrin polyclonal antibody (PROTEINTECK) and mouse anti-duck-annexin A2 polyclonal antibody prepared in our laboratory described as above. Thirty microgram proteins from each sample were separated in 12% SDS-PAGE gels and transferred to PVDF membranes using the transfer system (BioRad). The blots were blocked with 5% nonfat milk for 2 h at room temperature and incubated at 4°C overnight with 1:200-1:500 dilution of primary antibody. The blots were then washed four times with PBS containing 0.1% Tween-20, and incubated with the appropriate horseradish peroxidase-conjugated secondary antibody (Santa Cruz Biotechnology) 1 hour at room temperature. After washed four times with PBS containing 0.1% Tween-20, the bands were developed with ECL detection reagent (Pierce). The same amount protein of each sample was applied to a parallel SDS-PAGE gel and stained with Coomassie brilliant blue.

## List of abbreviations

2-DE: two-dimensional polyacrylamide gel electrophoresis; ACN: acetonitrile; DHBV: Duck hepatitis B virus; DAPI: 4',6'-diamidino-2-phenylindole; DTT: dithiothreitol; FBS: fetal bovine serum; GAPDH: glyceraldehyde-3-phosphate dehydrogenase; HBV: hepatitis B virus; HCV: hepatitis C virus; HCMV: human cytomegalovirus; HIV: human immunodeficiency virus; Hsp: heat shock protein; HSV: herpes simplex virus; IEF: isoelectric focusing; IPG: immobilized pH gradient; MOI: multiplicity of infection; MS: mass spectrometry; MS/MS: tandem mass spectrometry, PBS: phosphate-buffered saline; PCR: polymerase chain reaction; PDHs: Primary duck hepatocytes; TCP1: t-complex polypeptide 1; WHBV: Woodchuck hepatitis B virus.

## Competing interests

The authors declare that they have no competing interests.

## Authors' contributions

DQ was responsible for the conception and design of the study. YZ were responsible for PDHs culture, virus infection and sample preparation. HB and SQ carried out the 2-DE gels experiments, image analysis and excised the protein spots. XZ performed the mass spectrometric analyses. YZ and LY confirmed the differential expression by Western blot. YZ and HB carried out the analysis and interpretation of data. DQ, YZ and HB wrote the manuscript. BX, SZ, QL, RY, TZ, PY have been involved in drafting the manuscript or revising it critically for important content. All authors read and approved the final manuscript.

## Supplementary Material

Additional File 1 **Detection of DHBV replicative intermediates in PDHs**. DHBV DNA in PDHs was detected by Southern blot hybridization with an alpha-^32^P-dCTP labeled DHBV-specific probe. Click here for file

Additional File 2 **Detection of DHBV DNA in the supernatant of PDHs**. Viral genomes in the supernatant of DHBV infected PDHs were quantified by real time PCR. Click here for file

Additional File 3 **The numbers of differentially expressed protein spots detected by 2-DE**. The numbers of differentially expressed protein spots revealed by 2-DE at 24, 72, 120 h post-DHBV infection were listed. Click here for file

Additional File 4 **Differentially expressed protein spots in DHBV-infected PDHs not identified by MALDI-TOF/TOF**. The expression ratio between DHBV infected and uninfected PDHs of unidentified spots underlined in Figure 2 were listed. Click here for file

Additional File 5 **Peptides of differentially expressed proteins in DHBV-infected PDHs identified by MALDI-TOF/TOF**. Peptides count and peptides identified were listed. Click here for file
