# Supplementary material for: Proteomic analysis of primary duck hepatocytes infected with duck hepatitis B virus
Source: Proteome Sci. 2010 Jun 7;8:28. doi: 10.1186/1477-5956-8-28 (PMC2904733; doi:10.1186/1477-5956-8-28)
Supplement: Additional File 1 — Detection of DHBV replicative intermediates in PDHs. DHBV DNA in PDHs was detected by Southern blot hybridization with an alpha-32P-dCTP labeled DHBV-specific probe. [file 1477-5956-8-28-S1.DOC]

**Additional File 1.**


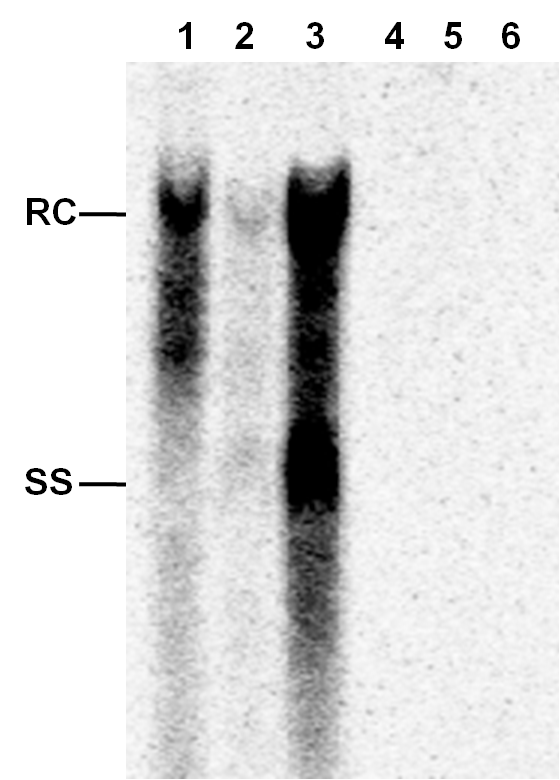


**Detection of DHBV replicative intermediates in PDHs.** PDHs infected with DHBV were harvested at 24, 72, 120 h post-infection and DHBV DNA was detected by Southern blot hybridization using an alpha-^32^P-dCTP labeled DHBV-specific probe (from lane 1-3, Lane 4-6 represent DNA from uninfected PDHs). The positions of relaxed circular (RC) and single stranded (SS) forms of intracellular viral DNA are indicated. The signal of 24 h is derived from input virus which lacks single stranded forms of DHBV.
