# Supplementary material for: Proteomic analysis of primary duck hepatocytes infected with duck hepatitis B virus
Source: Proteome Sci. 2010 Jun 7;8:28. doi: 10.1186/1477-5956-8-28 (PMC2904733; doi:10.1186/1477-5956-8-28)
Supplement: Additional File 2 — Detection of DHBV DNA in the supernatant of PDHs. Viral genomes in the supernatant of DHBV infected PDHs were quantified by real time PCR. [file 1477-5956-8-28-S2.DOC]

**Additional File 2.**


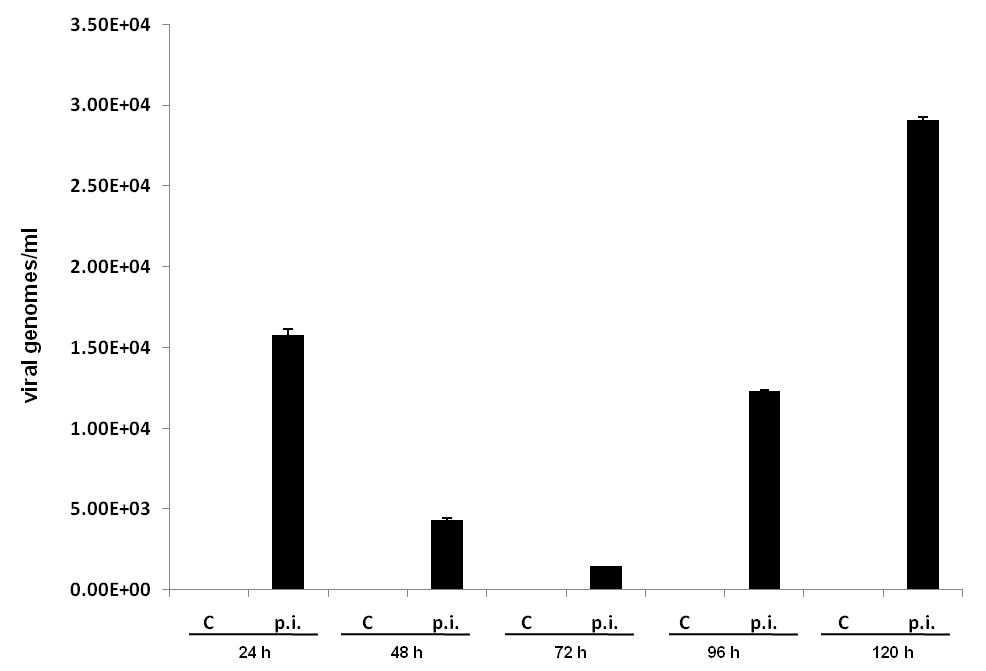


**Detection of DHBV DNA in the supernatant of PDHs.** DHBV DNA in the supernatant of infected PDHs was measured by real time PCR with DHBV specific primes (DHBV qPCR-f: 5'-TACATTGCTGTTGTCGTGTG-3', DHBV qPCR-r: 5'-ATTGGCTAAGGCTCTAGAAG-3', DHBV Probe: 5'-TGACTGTACCTTTGGTATGTACCATTG-3'). At least three samples were assayed for each time point. Secretion of DHBV particles was increased after 96 h while most of the viral paticles before 48 h were from the input virus demonstrated by Southern blot in Additional File 1.
