# Supplementary material for: Proteomic analysis of primary duck hepatocytes infected with duck hepatitis B virus
Source: Proteome Sci. 2010 Jun 7;8:28. doi: 10.1186/1477-5956-8-28 (PMC2904733; doi:10.1186/1477-5956-8-28)
Supplement: Additional File 3 — The numbers of differentially expressed protein spots detected by 2-DE. The numbers of differentially expressed protein spots revealed by 2-DE at 24, 72, 120 h post-DHBV infection were listed. [file 1477-5956-8-28-S3.DOC]

**Additional File 3.**

**The numbers of differentially expressed protein spots detected by 2-DE**

|  | The numbers of differentially expressed protein spots^a^ | |
| --- | --- | --- |
|  | Increased post-infection^b^ | Decreased post-infection |
| 24 h | 10 | 12 |
| 72 h | 22 | 11 |
| 120 h | 21 | 15 |

1. Differentially expressed spots with *p*-values less than 0.05 with at least a 1.5-fold difference in percentage of the volume by ImageMaster showed in Figure 2.
2. DHBV infected PDHs versus uninfected PDHs.
