# Supplementary material for: Proteomic analysis of primary duck hepatocytes infected with duck hepatitis B virus
Source: Proteome Sci. 2010 Jun 7;8:28. doi: 10.1186/1477-5956-8-28 (PMC2904733; doi:10.1186/1477-5956-8-28)
Supplement: Additional File 4 — Differentially expressed protein spots in DHBV-infected PDHs not identified by MALDI-TOF/TOF. The expression ratio between DHBV infected and uninfected PDHs of unidentified spots underlined in Figure 2 were listed. [file 1477-5956-8-28-S4.DOC]

**Additional File 4.**

**Differentially expressed protein spots in DHBV-infected PDHs not identified by MALDI-TOF/TOF**

| Time | Spot no.^a^ | Ratio: infected/uninfected^b^ |
| --- | --- | --- |
| 24 h | 232 | 2.5 |
|  | 761 | 1.5 |
|  | 807 | 0.67 |
|  | 1012 | 0.5 |
|  | 1095 | N/A^c^ |
| 72 h | 62 | 2 |
|  | 253 | 2 |
|  | 787 | 0.33 |
|  | 918 | 1.5 |
|  | 1098 | A/N |
|  | 1152 | N/A |
|  | 1156 | 0.5 |
| 120 h | 47p.i.^d^ | 2 |
|  | 387 | 2 |
|  | 583 | 1.5 |
|  | 654 | 1.5 |
|  | 657 | N/A |
|  | 663 | 0.33 |
|  | 851 | N/A |
|  | 862 | 0.33 |
|  | 882 | 1.67 |
|  | 893 | 3 |
|  | 936p.i. | 3 |
|  | 1006 | 0.67 |
|  | 1074 | N/A |

a) Spot numbers correspond to the numbers in Figure 2.

b) Ratio: the standardized ratio between DHBV infected PDHs versus uninfected PDHs.

c) A represents the spot on one of the gels was detectable, N represents the spot on one of the gels was too weak to detect.

d) p.i.: post-infection.
