# Supplementary material for: Proteomic analysis of primary duck hepatocytes infected with duck hepatitis B virus
Source: Proteome Sci. 2010 Jun 7;8:28. doi: 10.1186/1477-5956-8-28 (PMC2904733; doi:10.1186/1477-5956-8-28)
Supplement: Additional File 5 — Peptides of differentially expressed proteins in DHBV-infected PDHs identified by MALDI-TOF/TOF. Peptides count and peptides identified were listed. [file 1477-5956-8-28-S5.DOC]

**Additional File 5.**

**Peptides of differentially expressed proteins in DHBV-infected PDHs identified by MALDI-TOF/TOF**

| No. ^a^ | Protein | Peptides Count | Peptides Identified^b^ |  |
| --- | --- | --- | --- | --- |
| 1 | Triosephosphate isomerase 1 | 19 | KFFVGGNWK GAFTGEISPAMIK HVFGESDELIGQK DIGAAWVILGHSER |  |
| 2 | Triosephosphate isomerase | 10 |  |  |
| 3 | Glyceraldehyde-3-phosphate dehydrogenase(GAPDH) | 11 | LVSWYDNEYGYSNR |  |
| 4 | Glyceraldehyde-3-phosphate dehydrogenase(GAPDH) | 9 | LVSWYDNEYGYSNR |  |
| 5 | Glyceraldehyde-3-phosphate dehydrogenase(GAPDH) | 11 | LVSWYDNEYGYSNR |  |
| 6 | Glyceraldehyde-3-phosphate dehydrogenase(GAPDH) | 8 | LVSWYDNEYGYSNR |  |
| 7 | Phosphoglycerate kinase 1 | 19 | LGDVYVNDAFGTAHR ALESPERPFLAILGGAK |  |
| 8 | Phosphoglycerate mutase 1 | 5 | HYGALTGLNK VLIAAHGNSLR |  |
| 9 | Alpha-enolase | 11 | YISPDQLADLYK |  |
| 10 | Alpha-enolase | 22 | LAQSNGWGVMVSHR VVIGMDVAASEFYR AAVPSGASTGIYEALELR AGYSDKVVIGMDVAASEFYR |  |
| 11 | Aconitase 2, mitochondrial | 18 | EGWPVDIR FTITPGSEQIR NALTQEFGPVPDTAR WAVIGDENYGEGSSR |  |
| 12 | ATP5A1 | 17 | VLSIGDGIAR AVDSLVPIGR VGLKAPGIIPR TSIAIDTIINQKR TGAIVDVPVGEELLGR |  |
| 13 | ATP5A1 protein | 11 | GIRPAINVGLSVSR EVAAFAQFGSDLDAATQQLLNR |  |
| 14 | Chain E, Chicken Cytochrome Bc1 Complex Inhibited By An Iodinated Analogue Of The Polyketide Crocacin-D | 5 | DPQHDLDR VHNDVTVPDFSAYR |  |
| 15 | similar to ubiquinol-cytochrome c reductase | 6 | ADLASYIDTHFKAPR |  |
| 16 | unnamed protein product | 10 | HGGTIPVVPTAEFQDR IIAEGANGPTTPEADKIFLER |  |
| 17 | similar to betaine homocysteine methyl transferase | 12 | AIAEELAPER AGPWTPEATVEHPEAVR |  |
| 18 | Pterin-4 alpha-carbinolamine dehydratase | 7 | EQLLPNLR AVGWNEVEGR |  |
| 19 | similar to Urocanase domain containing 1 | 13 | FFPDIEMR GLPLNPLPENR |  |
| 20 | similar to Urocanase domain containing 1 | 21 | FFPDIEMR LVLEFAEELR LYGHIYMYR |  |
| 21 | similar to 3-mercaptopyruvate sulfurtransferase | 9 | VVDASWYLPK TYEDILDNLDSHR |  |
| 22 | Aldehyde dehydrogenase 4A1 | 6 | KEWDLKPVQDR STGAVVAQQPFGGSR |  |
| 23 | Transgelin | 7 | QMEQIAQFLK |  |
| 24 | Vinculin | 16 | AIPDLTAPVSAVQAAVSNLVR |  |
| 25 | Destrin | 11 | YALYDASFETK |  |
| 26 | Lamin A | 26 | NIYSEELR SLELENAGLR |  |
| 27 | Lamin A | 24 | NIYSEELR |  |
| 28 | similar to Myosin regulatory light chain 2,nonsarcomeric (Myosin RLC) isoform 1 | 7 | LNGTDPEDVIR EAFNMIDQNR GNFNYIEFTR FTDEEVDELYR |  |
| 29 | similar to HSPC162(dynein, light chain) | 4 | DIDPQNDLTFLR |  |
| 30 | Collapsin response mediator protein-2B | 12 | IFNLYPR KPFPDFVYK MVIPGGIDVHTR IVLEDGNLHVTEGSGR |  |
| 31 | Cofilin-2 | 3 | LGGNVVVSLEGKPL QILVGDIGDTVEDPYTAFVK |  |
| 32 | Gelsolin | 8 | EPGLQIWR AGKEPGLQIWR |  |
| 33 | similar to LIM protein | 7 | DFEQPLTISR |  |
| 34 | beta-actin | 13 | GYSFTTTAER QEYDESGPSIVHR LDLAGRDLTDYLMK SYELPDGQVITIGNER DLYANTVLSGGTTMYPGIADR |  |
| 35 | similar to heat shock 70kDa protein 8 isoform 2 isoform 2 | 5 | DAGTIAGLNVLR TVTNAVVTVPAYFNDSQR |  |
| 36 | Chain A, T13g Mutant Of The Atpase Fragment Of Bovine Hsc70 | 12 | DAGTIAGLNVLR TTPSYVAFTDTER TVTNAVVTVPAYFNDSQR |  |
| 37 | Heat shock 70kDa protein 5 precursor | 36 | ITPSYVAFTPEGER IINEPTAAAIAYGLDKR VTHAVVTVPAYFNDAQR DNHLLGTFDLTGIPPAPR |  |
| 38 | Heat shock 70kDa protein 5 precursor | 30 | ITPSYVAFTPEGER IINEPTAAAIAYGLDKR VTHAVVTVPAYFNDAQR DNHLLGTFDLTGIPPAPR |  |
| 39 | Chaperonin containing TCP1, subunit 6A (zeta 1) | 17 | EGIVALRR GLVLDHGAR QADLYISEGLHPR |  |
| 40 | 58kDa glucose regulated protein precursor | 16 | GFPTIYFAPAGK EVSDFISYLKR FLQDYFDGNLKK MDATANDVPSPYEVR |  |
| 41 | Mn superoxide dismutase | 7 | DFGSFANFK HHAAYVNNLNVAEEK FNGGGHINHTIFWTNLSPNGGGEPK |  |
| 42 | similar to antioxidant protein isoform 2 | 9 | HLSVNDLPVGR GLFIIDPNGVIK DYGVLLEGPGLALR |  |
| 43 | LOC496089 protein | 6 | QITMNDLPVGR DYGVYLEDQGHTLR |  |
| 44 | Elongation factor 2 | 12 | SDPVVSYR TGTITTFEHAHNMR TGTITTFEHAHNMR |  |
| 45 | Elongation factor 2 | 3 | AYLPVNESFGFTADLR GHVFEESQVAGTPMFVVK |  |
| 46 | Cathepsin B | 4 | SGVYQHVSGEQVGGHAIR |  |
| 47 | Ubiquitin carboxyl-terminal esterase L1 (ubiquitin thiolesterase) | 5 | FLDETADLSPEER MPFPVNHGTSSDDLLLK |  |
| 48 | Ovotransferrin | 12 | YYGYTGALR LKPIAAEVYER VPTHAVVARPEK |  |
| 49 | Annexin A2 | 13 | WINIMTER QDIAFAYQR |  |
| 50 | Annexin A2 | 8 | QDIAFAYQR GVDEVTIINILTNR LSLEGDHSLPPSAYATVK AYSNFDADRDAAALEAAIK |  |
| 51 | Annexin A5 | 16 |  |  |

a) Spots are named according to Figure 2.

b) The peptides identified by MALDI-TOF/TOF with statistically significant ion score (confidence interval, 95%).
